# Klotho-derived peptide 1 inhibits cellular senescence in the fibrotic kidney by restoring Klotho expression via posttranscriptional regulation

**DOI:** 10.7150/thno.89105

**Published:** 2024-01-01

**Authors:** Xiaoyao Zhang, Li Li, Huishi Tan, Xue Hong, Qian Yuan, Fan Fan Hou, Lili Zhou, Youhua Liu

**Affiliations:** 1 Division of Nephrology, Nanfang Hospital, Southern Medical University, Guangzhou, China.; 2 State Key Laboratory of Organ Failure Research, National Clinical Research Center of Kidney Disease, Guangdong Provincial Institute of Nephrology, Guangzhou, China.

**Keywords:** Klotho, cellular senescence, miRNA-223-3p, lncRNA-TUG1, kidney fibrosis, chronic kidney disease

## Abstract

**Background:** Klotho deficiency is a common feature of premature aging and chronic kidney disease (CKD). As such, restoring Klotho expression could be a logic strategy for protecting against various nephropathies. In this study, we demonstrate that KP1, a Klotho-derived peptide, inhibits cellular senescence by restoring endogenous Klotho expression.

**Methods:** The effects of KP1 on cellular senescence and Klotho expression were assessed in mouse models of CKD. RNA-sequencing was employed to identify the microRNA involved in regulating Klotho by KP1. Gain- or loss-of-function approaches were used to assess the role of miR-223-3p and IncRNA-TUG1 in regulating Klotho and cellular senescence.

**Results:** KP1 inhibited senescence markers p21, p16 and γ-H2AX in tubular epithelial cells of diseased kidneys, which was associated with its restoration of Klotho expression at the posttranscriptional level. Profiling of kidney microRNAs by RNA sequencing identified miR-223-3p that bound to Klotho mRNA and inhibited its protein expression. Overexpression of miR-223-3p inhibited Klotho and induced p21, p16 and γ-H2AX, which were negated by KP1. Conversely, inhibition of miR-223-3p restored Klotho expression, inhibited cellular senescence. Furthermore, miR-223-3p interacted with lncRNA-TUG1 and inhibited its expression. Knockdown of lncRNA-TUG1 increased miR-223-3p, aggravated Klotho loss and worsened cellular senescence, whereas KP1 mitigated all these changes.

**Conclusion:** These studies demonstrate that KP1 inhibits cellular senescence and induces Klotho expression via posttranscriptional regulation mediated by miR-223-3p and lncRNA-TUG1. By restoring endogenous Klotho, KP1 elicits a broad spectrum of protective actions and could serve as a promising therapeutic agent for fibrotic kidney disorders.

## Introduction

Chronic kidney disease (CKD) is a condition of kidney insufficiency lasting for more than 3 months, regardless of the initial causes 1. CKD is often characterized by increased cellular senescence, epigenetic reprogramming, sustained fibroblast activation and relentless extracellular matrix (ECM) production 2. Evidence suggests that CKD and aged kidney share many striking similarities 3-6. As such, CKD is now considered as a state of premature and accelerated kidney aging 7, 8. Many cellular senescence markers are upregulated in CKD, such as the cyclin-dependent kinase inhibitors including p21^cip1^ (p21) 9, 10 and p16^Ink4a^ (p16) 10, 11, phosphorylation of the Ser-139 residue of the histone variant H2AX (γ-H2AX) 12, 13 and senescence-associated β-galactosidase (SA-β-GAL) 9, 11, 14. It becomes clear that the dysregulation of anti-aging program after kidney injury plays a crucial role in the pathogenesis of CKD 4, 15, 16.

The anti-aging protein Klotho belongs to a small family of single-span transmembrane proteins consisting of α and β isoforms 17. The α-Klotho, referred as Klotho hereafter, is abundantly expressed in healthy kidneys, especially in the distal tubules 17. Klotho exists as membranous and soluble forms. Membranous Klotho (mKlotho) is a co-receptor of fibroblast growth factor 23 and regulates calcium and phosphorus homeostasis 18, 19, whereas soluble Klotho (sKlotho) is present in the circulation and acts as an endocrine factor 20, 21. Kidney injury causes a marked loss of Klotho expression, and Klotho deficiency can lead to hyperphosphatemia, arteriosclerosis, inflammation, bone mineral disorders, tubular cell senescence and renal fibrosis, thereby further accelerating the progression of CKD 22. Klotho has been postulated as a potential biomarker for the diagnosis and prognosis of kidney disorders and a therapeutic target 22-25. However, it remains to be evaluated clinically whether exogenous Klotho is beneficial for CKD patients.

Because Klotho is a large transmembrane protein, it is difficult and costly to produce it in large quantities. We recently report the discovery of the Klotho-derived peptide 1 (KP1), a 30-amino acid peptide derived from human Klotho protein. KP1 has been shown to attenuate kidney fibrosis by binding to TGF-β receptor 2 (TβR2) and inhibiting the TGF-β/Smad3 signaling 26. These observations indicate that KP1 may recapitulate the anti-fibrotic function of Klotho protein. Furthermore, as KP1 can be synthesized chemically and cost-effectively, it holds the potential to substitute Klotho for clinical translation.

To further investigate the role of KP1 in protecting kidney, we examined its effects on cellular senescence, a critical event in the pathogenesis of CKD 26. We found that KP1 inhibited kidney tubular cell senescence, which was associated with its ability to restore Klotho expression at the posttranscriptional level. Our studies implicate miRNA-223-3p and long non-coding RNA (lncRNA)-TUG1 in mediating KP1 regulation of Klotho expression. Therefore, restoration of Klotho expression via epigenetic regulation could be an important mechanism of KP1 action.

## Methods

### Peptide synthesis

The KP1 was described previously 26 and synthesized by GenScript (Piscataway, NJ) with a purity of > 95%. KP1 was dissolved in 0.01 M acetic acid solution at a concentration of 10 μg/μL.

### Animal models

Animal studies were approved by the Animal Ethics Committee at the Nanfang Hospital, Southern Medical University. Male C57BL/6 mice aged 8-10 weeks were purchased from Beijing Vital River Laboratory Animal Technology Co. For UUO model, mice were anesthetized and their left ureter ligated 27. The sham group underwent exposure of the left kidney without ligation of the ureter. On the 7th day after surgery, mice were euthanized and left kidney tissue collected. For UIRI model, mice were anesthetized and renal artery was clamped for 28 min before releasing it. During ischemia, the body temperature of mice was maintained at 37.5 ℃. On day 10 after surgery, contralateral normal kidney was removed. Kidney tissue and blood were collected on day 11 after surgery. The sham group underwent all procedures except for clamping of the renal artery.

### Injection of KP1, plasmid and antagomir

KP1 was administered via daily intravenous injection at the dose of 1 mg/kg/day starting from day 1 after UUO and day 4 after UIRI, respectively. We chose to start the KP1 treatment at 4 days after IRI to avoid its effect on acute kidney injury (AKI) phase of this model. KP1 was enriched in the injured kidneys after intravenous injection 26. The expression plasmids used in this study were customarily made by GenePharma (Suzhou, China) and prepared using QIAfilter Plasmid Midi and Maxi Kits (QIAGEN, Hilden, Germany) according to the manufacturer's instructions. Plasmids were injected intravenously at a dose of 2.5 mg/kg (diluted with a total volume of 2 mL of saline) on day 1 after UUO and day 4 after UIRI. The antagomirs were designed and produced by Tsingke Biotechnology (Beijing, China). Antagomirs were administered every other day starting on day 1 after UUO and day 4 after UIRI, respectively.

### Cell culture and treatment

Human kidney proximal tubular cells (HK-2) and human embryonic kidney cells (HEK-293T) were purchased from the American Type Culture Collection (Manassas, VA). After 24 h of serum starvation, HK-2 cells were pretreated with KP1 (10 ng/mL), SB431542 (10 μM) and SIS3 (10 μM) for 1 h and then treated with TGF-β1 (2 ng/mL) for 45 min or 48 h. In some experiments, KP1 (10 ng/mL), TGF-β1 (2 ng/mL) and actinomycin D (5 μg/mL) or cycloheximide (5 μg/mL) were added and cells collected at different time points as indicated.

### Mouse primary proximal tubular epithelial cells

Mouse primary proximal tubular epithelial cells were isolated and cultured as previously described 28. Briefly, the cortical part of mouse kidneys was minced, then digested in pre-warmed 1 mg/mL collagenase 1 for 60 min at 37 °C, after which the mashed tissue was sieved in DMEM/F-12. Tubules were suspended in DMEM/F-12 supplemented with 10% bovine calf serum, 50 U/mL penicillin and 50 mg/mL streptomycin. Cells were cultivated for 4-8 days until they reached 60%-80% confluency. Tubular epithelial cells were characterized by morphology, positive staining for E-cadherin and negative staining for vimentin, respectively. The culture medium was changed on day 2 and 5, and then every 3 days.

### Western blot analysis

Proteins were extracted from kidney tissue and HK-2 cells and separated using sodium dodecyl sulfate-polyacrylamide gel electrophoresis (SDS-PAGE). The proteins were then transferred to a polyvinylidene fluoride (PVDF) microporous membrane, and then blocked with 5% non-fat milk before incubation with the appropriate primary antibodies as indicated. The protein bands were visualized by SuperEnhanced chemiluminescence detection reagents. The results were scanned and the intensity of the bands was analyzed and quantified using ImageJ software (NIH, Bethesda, MD) and normalized to appropriate internal control. The sources of antibodies used are listed in supplementary Table S1.

### qRT-PCR

Total RNA was extracted from kidney tissue and HK-2 cells using TRIzol Reagent Kit (Invitrogen, Carlsbad, CA). For miR-223-3p, total RNA was reverse transcribed to cDNA using the Mir-X^TM^ miRNA First-Strand Synthesis Kit (Takara Bio Inc., CA, USA) according to the instructions of the manufacturer. For mRNA and lncRNA, total RNA was reverse transcribed to cDNA using the GoScript Reverse Transcription System Kit (Promega, Madison, WI). The sequences of specific primers are given in supplementary Table S2. The miRNA, lncRNA and mRNA were quantitatively analyzed using SYBR Green PCR Master Mix (Promega, USA), while small nuclear U6 was used as an internal control for miR-223-3p and β-actin used as an internal control for mRNA and lncRNA, respectively.

### miRNA sequencing and bioinformatics analysis

Three groups of mice (sham, UIRI, and UIRI + KP1) were used, with three biological replicates per group. Total RNA was extracted using TRIzol Reagent (Life Technologies). The miRNA sequencing was customarily carried out by Beijing Biomarker Technologies Co. The sequencing library was generated using Hieff NGS Ultima Dual-mode mRNA Library Prep Kit for Illumina (Yeasen Biotechnology, Shanghai, China), and sequenced on the Illumina NovaSeq platform. The data were analyzed using the bioinformatics analysis platform BMKCloud. Differential expression analysis was performed between two groups using DESeq2. Adjusted P values were obtained using the Benjamini and Hochberg method to control the false discovery rate. Genes with adjusted *P* values < 0.01 and fold change ≥ 2 from DESeq2 analysis were designated as differentially expressed.

We predicted the miRNAs that might bind to the Klotho mRNA 3'-UTR using TargetScan, performed sequence alignment using NCBI Blast to ensure that the 2nd-7th nucleotides of the 5' end of the selected miRNAs were complementary to the Klotho mRNA. We then validated the miRNAs that showed large expression differences in the sequencing data. We used RNAhybrid to predict whether each lncRNA could bind specifically to miR-223-3p and their minimum free energy (mfe), and selected the lncRNAs with mfe less than -20 kcal/mol and complementary to the 2nd-7th nucleotides of the 5' end of miR-223-3p for validation.

### Cell transfection

The miR-223-3p mimics and inhibitor, lncRNA-TUG1 siRNA were obtained from GenePharma (Suzhou, China). HK-2 cells were cultured in 6-well plates for 16-24 h before transfection. Prior to transfection, cell culture medium was replaced with serum-reduced medium (Opti-MEM I; Invitrogen, Carlsbad, CA). The transfection was carried out with Lipofectamine 2000 (Invitrogen) according to the manufacturer's instructions. The mimics (20 nM) or inhibitor (100 nM) or siRNA (20 nM)/Lipofectamine 2000 mixture were added to HK-2 cells and incubated for 6-8 h before being replaced with 10% FBS medium and cultured for another 48 h. The sequences of siRNA-TUG1-homo, hsa-miR-223-3p mimics, hsa-miR-223-3p inhibitor, hsa-miRNA negative control (NC) are presented in supplementary Table S3 and S4.

### Luciferase reporter assay

The Dual-Luciferase pmirGLO plasmids used in this study were obtained from GenePharma. To verify the specific binding of miR-223-3p to the 3'-UTR of Klotho mRNA, we mutated the sequence of the putative binding site. The pmirGLO-h-KL-miR223-wt or pmirGLO-h-miR223-mut was mixed with miR-223-3p mimics or negative control (NC), respectively, and then mixed with Lipofectamine 2000 reagent. The mixture was transfected into HEK 293T cells. Luciferase activities were assessed after 24 h after transfection using Dual-Luciferase Reporter Assay System (Promega) according to the manufacturer's instructions. The relative luciferase activity (arbitrary units) after normalizing to *Renilla* luciferase was reported as fold induction over the controls. The specific binding of miR-223-3p to lncRNA-TUG1 was carried out in a similar way as described above, and pmirGLO-h-NR152868.2-miR223-wt plasmid and pmirGLO-h-NR152868.2-miR223-mut plasmids were used.

### Histology and immunohistochemical staining

Paraffin-embedded kidney sections were prepared and subjected to Masson's trichrome staining (MTS). After antigen unmasking, sections were incubated with primary antibodies overnight at 4℃, followed by incubation with biotinylated secondary antibodies and developed with amino ethyl carbazol (AEC) substrate. The tissue sections were counterstained with hematoxylin. Quantification of the images was carried out using Image Pro-Plus v. 6.0 software (Bethesda, MD). The sources of antibodies used were listed in supplementary Table S1.

### Detection of serum creatinine and blood urea nitrogen

Serum creatinine (Scr) and blood urea nitrogen (BUN) levels were determined by an automatic chemistry analyzer (AU480; Beckman 496 Coulter, Brea, CA). The levels of Scr and BUN were expressed as mg/dL.

### RNA fluorescence *in situ* hybridization

RNA fluorescence *in situ* hybridization (FISH) was carried out by using RNA FISH kit obtained from GenePharma, according to the protocols specified by the manufacturer. The Cy2-labeled mouse miR-223-3p probe, Cy3-labeled mouse lncRNA-TUG1 probe and Cy3-labeled mouse Klotho mRNA probe were also designed and produced by GenePharm. The specific experimental procedures were followed according to the protocol of the RNA FISH kit.

### Statistical analysis

All data examined were results of at least 3 independent repeated experiments in cell culture and 6 animals per group, and expressed as mean ± SEM. Statistical analysis of the data was carried out using GraphPad Prism 9.0.0 with t-tests for comparisons between 2 groups; one-way ANOVA for comparisons between multiple groups, followed by LSD/Dunnett tests. *P* < 0.05 was considered significant.

## Results

### KP1 inhibits cellular senescence and restores Klotho expression in the fibrotic kidney

**Figure 1:**
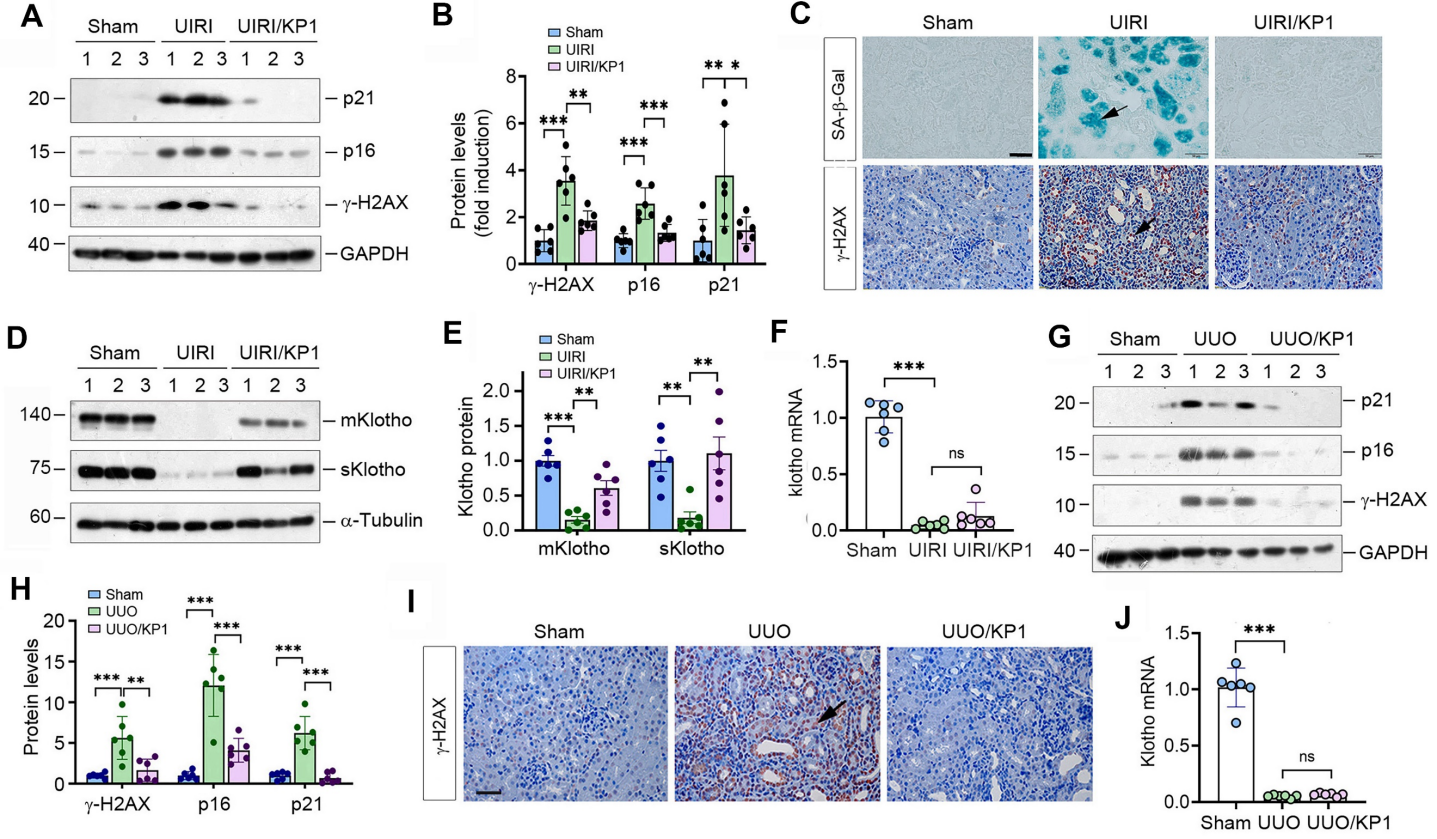
** KP1 inhibits cellular senescence and upregulates endogenous Klotho expression in fibrotic kidney.** (**A**, **B**) Western blotting analysis showed that KP1 abolished the induction of p21, p16 and γ-H2AX proteins in the kidneys of UIRI mice. (**C**) Representative micrographs demonstrated SA-β-Gal and γ-H2AX staining in various groups as indicated. Arrow indicated positive staining. Scale bar, 50 µm. (**D**,** E**) Western blotting analysis for mKlotho and sKlotho protein levels in UIRI mice after KP1 treatment. (**F**) qRT-PCR analysis of Klotho mRNA levels in UIRI mice after KP1 treatment. (**G**, **H**) Western blotting analysis for p21, p16 and r-H2AX protein levels in UUO mice after KP1 treatment. (**I**) Representative micrographs showedγ-H2AX staining in UUO mice after KP1 treatment. Arrow indicated positive staining. Scale bar, 50 µm. (**J**) qRT-PCR analysis of Klotho mRNA levels in UUO mice after KP1 treatment. ^*^P < 0.05, ^**^P < 0.01, ^**^P < 0.001 (n=6). ns, no statistical difference*.*

We first examined the effect of KP1 on cellular senescence in mouse model of CKD induced UIRI. As shown in Figure 1A and B, UIRI induced the expression of numerous cellular senescence markers such as p21, p16 and γ-H2AX in the fibrotic kidney, whereas KP1 abolished their induction. Staining for SA-β-Gal revealed abundant senescent cells in the tubular epithelium of UIRI kidney, whereas KP1 largely abolished it (Figure 1C). Immunostaining for γ-H2AX gave rise to the same results (Figure 1C).

To elucidate how KP1 inhibits cellular senescence, we examined the expression of endogenous Klotho. As shown in Figure 1D and E, UIRI caused drastic loss of both mKlotho and sKlotho in the kidney, whereas KP1 largely restored their expression. UIRI also caused a dramatic down-regulation of Klotho mRNA. However, KP1 did not affect the steady-state level of Klotho mRNA (Figure 1F).

To generalize these findings, we used another model of CKD induced by UUO. As shown in Figure 1G and H, KP1 also inhibited p21, p16 and γ-H2AX in UUO kidney. Immunostaining for γ-H2AX produced similar results (Figure 1I). KP1 also restored renal expression of mKlotho and sKlotho proteins (Figure S1) but did not affect its mRNA levels (Figure 1J). Collectively, it appears that KP1 inhibits cellular senescence in the fibrotic kidney, which is associated with the restoration of Klotho protein.

### KP1 induces Klotho expression without affecting its mRNA and protein stability

**Figure 2:**
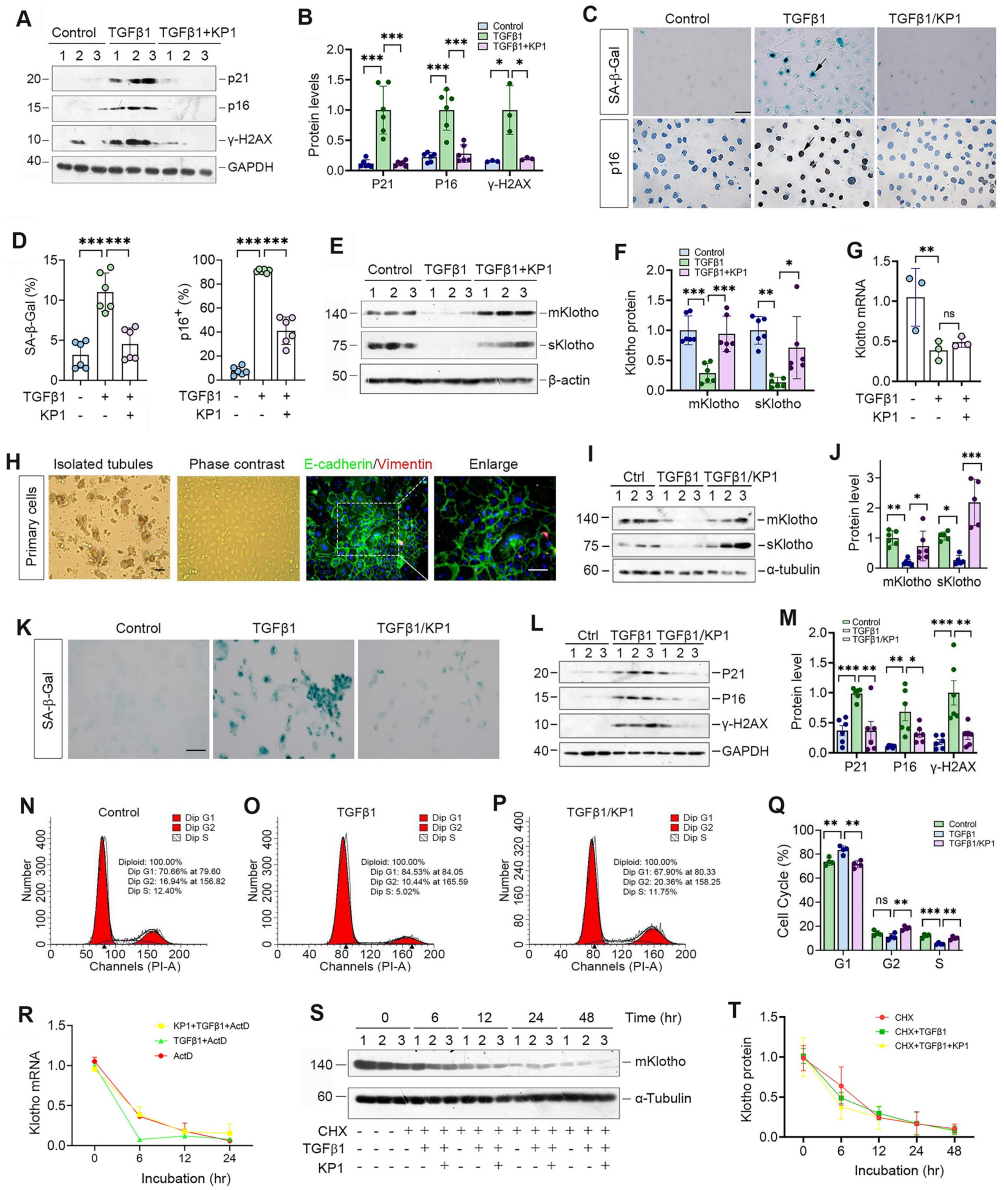
** KP1 alleviates tubular cell senescence and restores Klotho expression without affecting its mRNA and protein stability.** (**A**, **B**) Representative Western blot (**A**) and quantitative data (**B**) showed that KP1 abolished the induction of p21, p16 and γ-H2AX proteins in TGF-β1-stimulated human kidney proximal tubular cells (HK-2). (**C**) Representative micrographs showed the positive staining for SA-β-Gal and p16 in various groups as indicated. Scale bar, 50 µm. (**D**) Quantitative data of SA-β-Gal^+^ and p16^+^ cells in various groups as indicated. (**E**, **F**) Representative Western blot (**E**) and quantitative data (**F**) showed that KP1 restored mKlotho and sKlotho expression in HK-2 cells. (**G**) qRT-PCR analysis of Klotho mRNA levels in TGF-β1-stimulated HK-2 after KP1 treatment. (**H**) Culture and characterization of mouse primary proximal tubular epithelial cells. Freshly isolated proximal tubules and primary tubular epithelial cells (phase contrast) are shown. Primary cells were characterized by immunostaining with specific antibodies against E-cadherin (green) and vimentin (red), respectively. Scale bar, 50 µm. (**I**, **J**) Representative Western blot (**I**) and quantitative data (**J**) showed that KP1 restored mKlotho and sKlotho expression in mouse primary proximal tubular epithelial cells. (**K**) Representative micrographs showed the positive staining for SA-β-Gal in various groups as indicated. Scale bar, 50 µm. (**L**, **M**) Representative Western blot (**L**) and quantitative data (**M**) showed that KP1 abolished the induction of p21, p16 and γ-H2AX proteins in TGF-β1-stimulated mouse primary proximal tubular epithelial cells. (**N**-**P**) Representative flow cytometry analysis of mouse primary proximal tubular epithelial cell cycle after various treatments as indicated. (**Q**) Quantitative data of cell cycle in mouse primary proximal tubular epithelial cells after various treatments as indicated. (**R**) qRT-PCR analysis showed the steady-state levels of Klotho mRNA in HK-2 cells at different time points after treatment with Actinomycin D (ActD) (n=3). (**S**, **T**) Western blot analysis of mKlotho protein levels in HK-2 cells at different time points after cycloheximide (CHX) treatment (n=3). ^*^P < 0.05, ^**^P < 0.01, ^**^P < 0.001 (n=6). ns, no statistical difference*.*

We further examined the role of KP1 in regulating cellular senescence *in vitro*. As shown in Figure 2A and B, KP1 also inhibited p21, p16 and γ-H2AX expression in human kidney proximal tubular (HK-2) cells triggered by TGF-β1. Staining for SA-β-Gal and p16 gave rise to similar results (Figure 2C and D). Moreover, KP1 restored Klotho proteins repressed by TGF-β1 (Figure 2E and F). However, KP1 did not affect Klotho mRNA levels (Figure 2G), suggesting that it induces Klotho by a mechanism independent of transcriptional regulation.

We also assessed the ability of KP1 in blocking cellular senescence using primarily cultured mouse proximal tubular epithelial cells. These mouse primary kidney tubular epithelial cells were characterized by E-cadherin-positive and vimentin-negative staining (Figure 2H). As shown in Figure 2I and J, KP1 also restored mKlotho and sKlotho expression in mouse primary proximal tubular epithelial cells after TGF-β1 treatment. TGF-β1 also induced cellular senescence of primary tubular cells, as manifested by SA-β-Gal, p21, p16 and γ-H2AX induction, which were abolished by KP1 (Figure 2K-M). Cell cycle analysis by flow cytometry demonstrated that senescent primary tubular cells after TGF-β1 incubation were arrested at the G1 phase, which was negated by KP1 treatment (Figure 2N-Q).

We then examined whether KP1 affects Klotho mRNA stability. To this end, HK-2 cells were treated with actinomycin D (ActD) to block new mRNA synthesis. The steady-state levels of Klotho mRNA declined after ActD treatment. We found that KP1 did not affect Klotho mRNA levels at 12 and 24 h after incubation with TGF-β1 in the presence ActD, although there was a tendency of an increased Klotho mRNA level at 6 h (Figure 2R). We also assessed whether KP1 affects Klotho protein stability. For this purpose, HK-2 cells were treated with cycloheximide (CHX), which blocks new protein synthesis. As shown in Figure 2S and T, KP1 did not change Klotho protein abundance in the absence or presence of TGF-β1 at various time points. Collectively, these results suggest that KP1 induces Klotho protein without affecting its mRNA and protein stabilities.

### KP1 upregulates Klotho protein by inhibiting miR-223-3p

**Figure 3:**
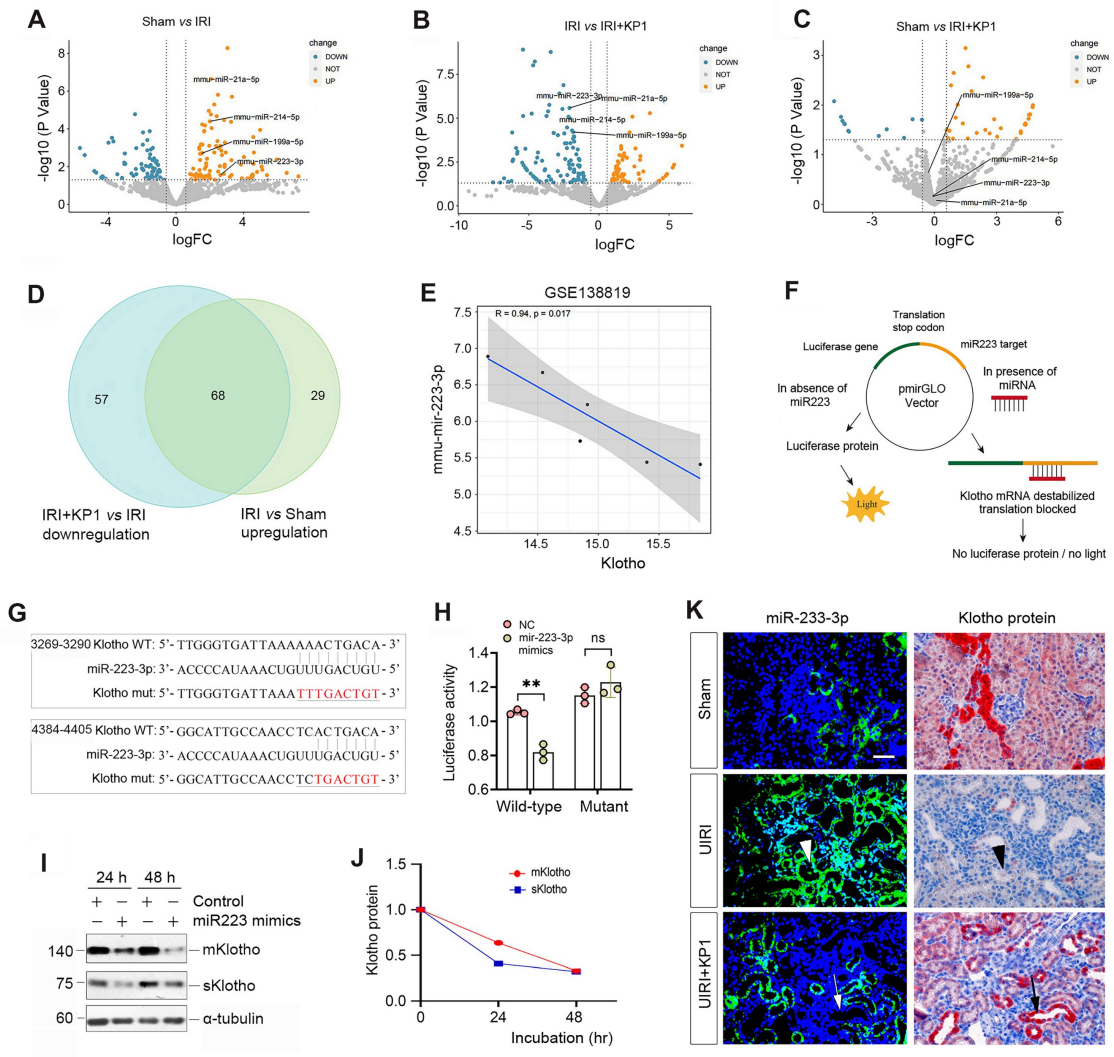
** KP1 up-regulates Klotho expression by inhibiting miR-223-3p.** (**A-C**) Volcano plots of the differentially expressed miRNAs in the kidneys of different groups as indicated. (**A**) Sham *versus* IRI, (**B**) IRI *versus* IRI+KP1, (**C**) Sham *versus* IRI+KP1 (n=3 biologically independent animals). (**D**) Venn diagram showed the differential expression of miRNAs in the kidneys of different groups. (**E**) Correlation analysis of Klotho and miR-223-3p expression levels in GSE138819 dataset (n=6 biologically independent animals). (**F**) Schematic diagram showed the function of miR-223-3p using the dual-luciferase reporter assay. (**G**) The sequence of the *Klotho* 3'-UTR with the wild-type (WT) or mutated binding site of miR-223-3p, which were inserted into a luciferase reporter. (**H**) Relative luciferase activity. HEK-293T cells were co-transfected with pmirGLO-h-KL-miR223-wt or pmirGLO-h-KL-miR223-mutant and miR-223-3p mimics or miR-negative control (NC) for 24 h. (**I, J**) Western blot analysis of mKlotho and sKlotho proteins in miR-223-3p mimics-transfected HK-2 cells for 24 h and 48 h, respectively. Representative Western blot (**I**) and quantitative data (**J**) are presented. (**K**) Localization of miR-223-3p and Klotho protein in the fibrotic kidney after UIRI. The expression of miR-223-3p was assessed by *in situ* hybridization (ISH), whereas the expression of Klotho protein was observed by immunohistochemical staining on serial sections. Arrowheads indicated renal tubules with high level of miR-223-3p and absence of Klotho, while arrows denoted the renal tubules with no expression of miR-223-3p and high level of Klotho protein. Scale bar, 50 µm.

As KP1 did not affect Klotho mRNA level and protein stability, this prompted us to explore whether KP1 regulates Klotho protein via microRNAs (miRNAs), a class of small noncoding RNAs known to play a role in the posttranscriptional regulation of gene expression. To this end, we investigated the differential expression of miRNAs in the kidney by RNA-sequencing. As shown in Figure 3A-C, there was substantial difference in miRNA profiling among sham, UIRI and UIRI plus KP1 groups. We identified 68 miRNAs which were upregulated in the UIRI kidney compared to sham controls, but returned to baseline after injection with KP1 (Figure 3D). Among these miRNAs, 10 of them were predicted by TargetScan program to specifically bind to the 3'-UTR of Klotho mRNA. In this group, miR-223-3p was particularly interesting, as it was induced in UIRI but returned to baseline after KP1 treatment (Figure 3A-C). By reanalyzing the GSE138819 dataset in the Gene Expression Omnibus (GEO) database from an independent study 29, miR-223-3p was also found to be negatively correlated with Klotho levels (Figure 3E), supporting the relevance of miR-223-3p to Klotho regulation.

To corroborate the regulatory role of miR-223-3p in Klotho expression, we carried out a dual-luciferase reporter assay (Figure 3F). Bioinformatics analyses predicted two putative binding sites of miR-223-3p in the 3-'UTR of Klotho mRNA (Figure 3G), and we constructed luciferase reporter plasmids containing wild-type or mutant Klotho mRNA sequence (Figure 3G). As shown in Figure 3H, transfection with miR-223-3p mimics reduced the luciferase activity of the reporter containing wild-type but not mutant Klotho mRNA sequence, indicating that miR-223-3p can specifically target Klotho mRNA, leading to its inhibition. To further confirm this, we transfected HK-2 cells with miR-223-3p for 24 and 48 h. As shown in Figure 3I and J, miR-223-3p inhibited the expression of Klotho proteins in HK-2 cells.

We next investigated the expression of miR-223-3p and its relationship with Klotho *in vivo*. As shown in Figure 3K, miR-223-3p was induced in renal tubular epithelium of UIRI kidney, as demonstrated by *in situ* hybridization. However, KP1 suppressed miR-223-3p expression (Figure 3K). Immunostaining for Klotho protein on serial sections demonstrated an inverse correlation between miR-223-3p and Klotho protein. In renal tubules with high level of miR-223-3p, Klotho protein was absent (Figure 3K, arrowheads); however, abundant Klotho was present in renal tubules lacking miR-223-3p expression (Figure 3K, arrows). These results suggest that KP1 could restore Klotho expression by inhibiting miR-223-3p *in vivo*.

### MiR-223-3p induces cellular senescence by targeted inhibition of Klotho *in vitro*

**Figure 4:**
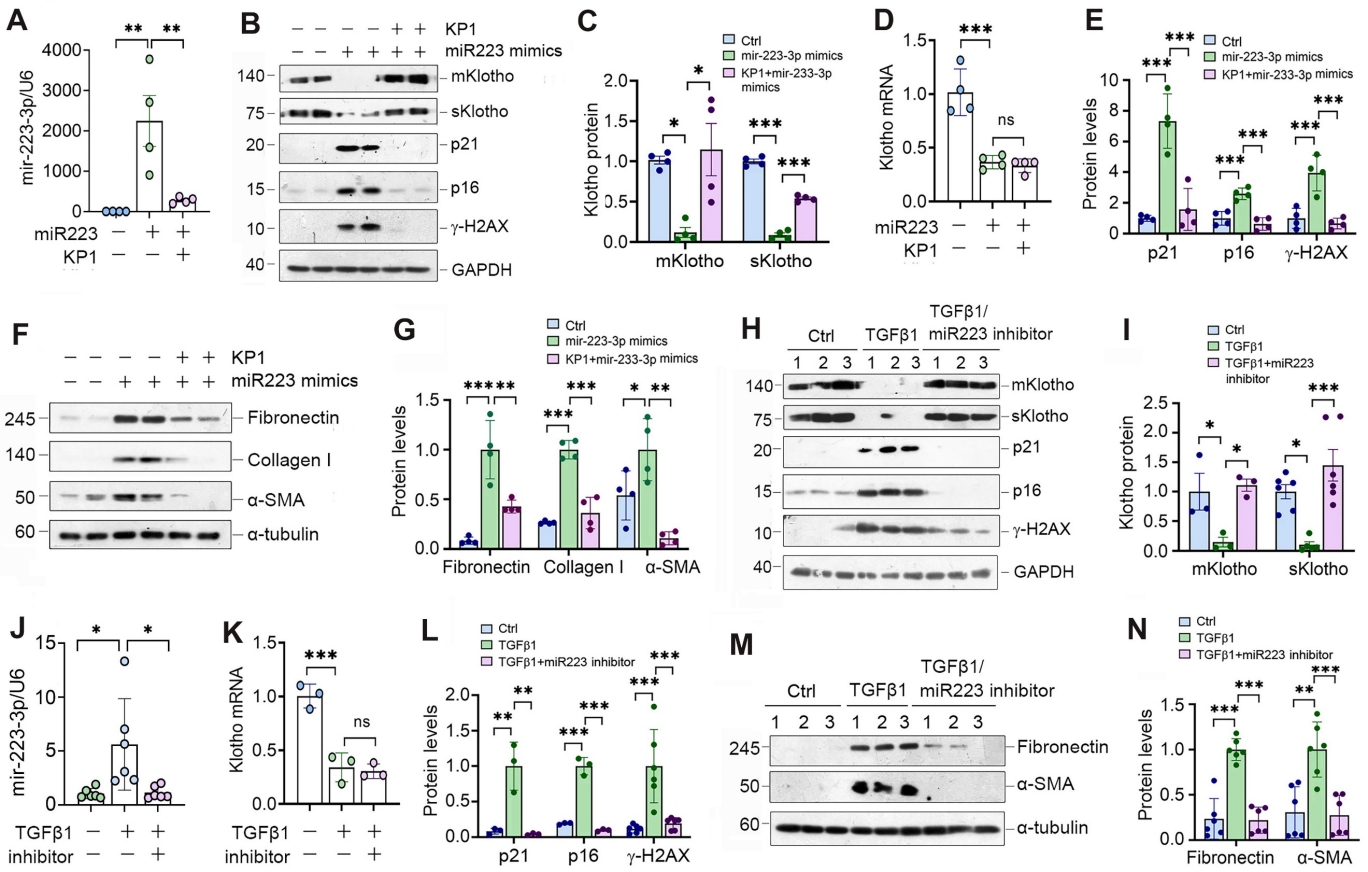
** miR-223-3p induces cellular senescence and negatively regulates Klotho *in vitro*.** (**A**) qRT-PCR analysis showed the miR-223-3p levels in HK-2 cells after transfection with miR-223-3p mimics in the absence or presence of KP1. (**B**) Representative Western blot analyses of mKlotho, sKlotho, p21, p16 and γ-H2AX proteins in HK-2 cells after transfection with miR-223-3p mimics in the absence or presence of KP1. (**C**, **D**) Quantitative data showed the levels of mKlotho and sKlotho (**C**) and Klotho mRNA (**D**) in different groups as indicated. (**E**) Quantitative data showed the levels of p21, p16 and γ-H2AX proteins in different groups as indicated. (**F**, **G**) Representative Western blot (**F**) and quantitative data (**G**) showed fibronectin, collagen I and α-SMA proteins after transfection with miR-223-3p mimics in HK-2 cells in the absence or presence of KP1. (**H**) Representative Western blot analyses of mKlotho, sKlotho, p21, p16 and γ-H2AX in TGF-β1-stimulated HK-2 cells in the absence or presence of miR-223-3p inhibitor. (**I**) Quantitative data of mKlotho and sKlotho protein levels in different groups as indicated. (**J**) qRT-PCR analysis showed the miR-223-3p levels in the TGF-β1-stimulated HK-2 cells in the absence or presence of miR-223-3p inhibitor. (**K**) qRT-PCR analysis of Klotho mRNA levels in TGF-β1-stimulated HK-2 cells in the absence or presence of miR-223-3p inhibitor. (**L**) Quantitative data of p21, p16 and γ-H2AX protein levels in different groups as indicated. (**M**, **N**) Representative Western blot (**M**) and quantitative data of fibronectin and α-SMA protein levels (**N**) in different groups as indicated. ^*^P < 0.05, ^**^P < 0.01, ^**^P < 0.001. ns, no statistical difference*.*

To explore the role of miR-223-3p in regulating cellular senescence, we manipulated its levels in HK-2 cells by overexpressing or inhibiting miR-223-3p (Figure 4A). As shown in Figure 4B and C, transfection of HK-2 cells with miR-223-3p mimics inhibited Klotho proteins, whereas KP1 restored their expression. However, KP1 did not alter Klotho mRNA level in HK-2 cells (Figure 4D). Overexpression of miR-223-3p alone could induce p21, p16 and γ-H2AX, which was negated by KP1 (Figure 4B and E). Furthermore, overexpression of miR-223-3p also induced fibronectin, collagen I and α-SMA expression in HK-2 cells, which was abolished by KP1 (Figure 4F and G).

We also used an opposite strategy by inhibiting miR-223-3p through transfection with its inhibitor. As presented in Figure 4H-J, inhibition of miR-223-3p could restore Klotho in TGF-β1-treated HK-2 cells but did not affect its mRNA levels. Inhibition of miR-223-3p also protected HK-2 cells from cellular senescence and fibrotic response caused by TGF-β1, as miR-223-3p inhibitor repressed p21, p16, γ-H2AX, fibronectin and α-SMA expression (Figure 4H, L-N).

### KP1 abolishes miR-223-3p-triggered Klotho reduction and alleviates kidney injury *in vivo*

**Figure 5:**
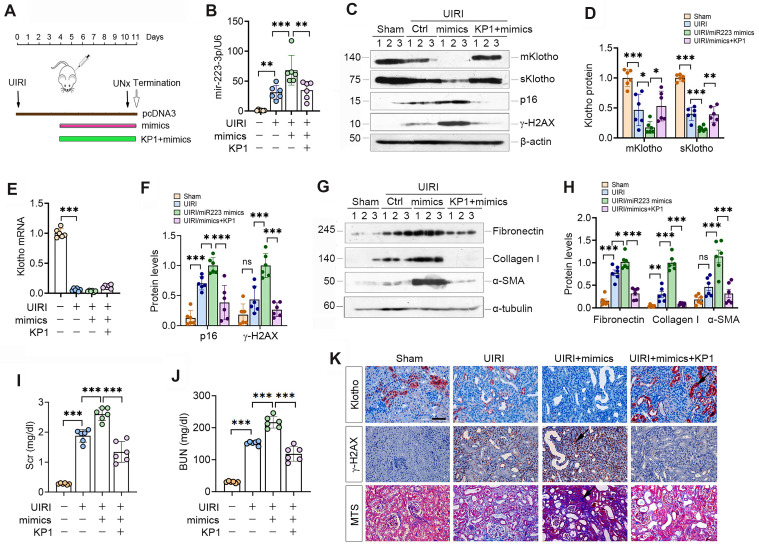
** KP1 restores Klotho expression and ameliorates nephropathy by repressing miR-223-3p *in vivo*.** (**A**) Schematic diagram shows experimental design with miR-223-3p overexpression and KP1 therapy. (**B**) qRT-PCR analysis showed the miR-223-3p levels after miR-223-3p-overexpression and KP1 treatment in UIRI mice. (**C**) Representative Western blot analyses of mKlotho, sKlotho, p16 and γ-H2AX proteins in different groups as indicated. (**D**) Quantitative data of mKlotho and sKlotho protein levels. (**E**) qRT-PCR analysis of Klotho mRNA levels in different groups as indicated. (**F**) Quantitative data of p16 and γ-H2AX protein levels. (**G**,** H**) Western blot analysis of fibronectin, collagen I and α-SMA proteins in different groups as indicated. (**I**, **J**) Graphic presentation shows the serum creatinine (Scr) and blood urea nitrogen (BUN) levels in different groups. (**K**) Representative immunostaining for Klotho and γ-H2AX, and MTS for collagens in UIRI mice. Scale bar, 50 µm. ^*^P < 0.05, ^**^P < 0.01, ^**^P < 0.001 (n=6). ns, no statistical difference*.*

We investigated the effect of miR-223-3p on Klotho expression in UIRI mice by intravenously injecting miR-223-3p expression plasmid and KP1 (Figure 5A). As shown in Figure 5B, miR-223-3p level was increased after injection of overexpression plasmid and decreased after KP1 treatment. Overexpression of miR-223-3p suppressed mKlotho and sKlotho in UIRI, while KP1 restored their expression (Figure 5C-D). However, Klotho mRNA levels did not substantially change after miR-223-3p overexpression or KP1 treatment (Figure 5E). Overexpression of miR-223-3p aggravated expression of p16, γ-H2AX, fibronectin, collagen I and α-SMA, all of which were inhibited by KP1 (Figure 5C, F-H). Scr and BUN assay showed that miR-223-3p overexpression worsened kidney function, whereas KP1 improved it (Figure 5I). Immunostaining gave rise to similar results (Figure 5P).

We also overexpressed miR-223-3p in UUO mice (Supplementary Figure S2). Similarly, miR-223-3p reduced Klotho protein but not its mRNA in UUO, while KP1 largely restored them (Figure S2A-D). Assessment of p21, p16, γ-H2AX, fibronectin, collagen I and α-SMA proteins indicated that miR-223-3p overexpression exacerbated cellular senescence and kidney fibrosis in UUO as well, while KP1 alleviated these lesions (Figure S2B, E-H).

### Antagonism of miR-223-3p restores Klotho and inhibits cellular senescence *in vivo*

**Figure 6:**
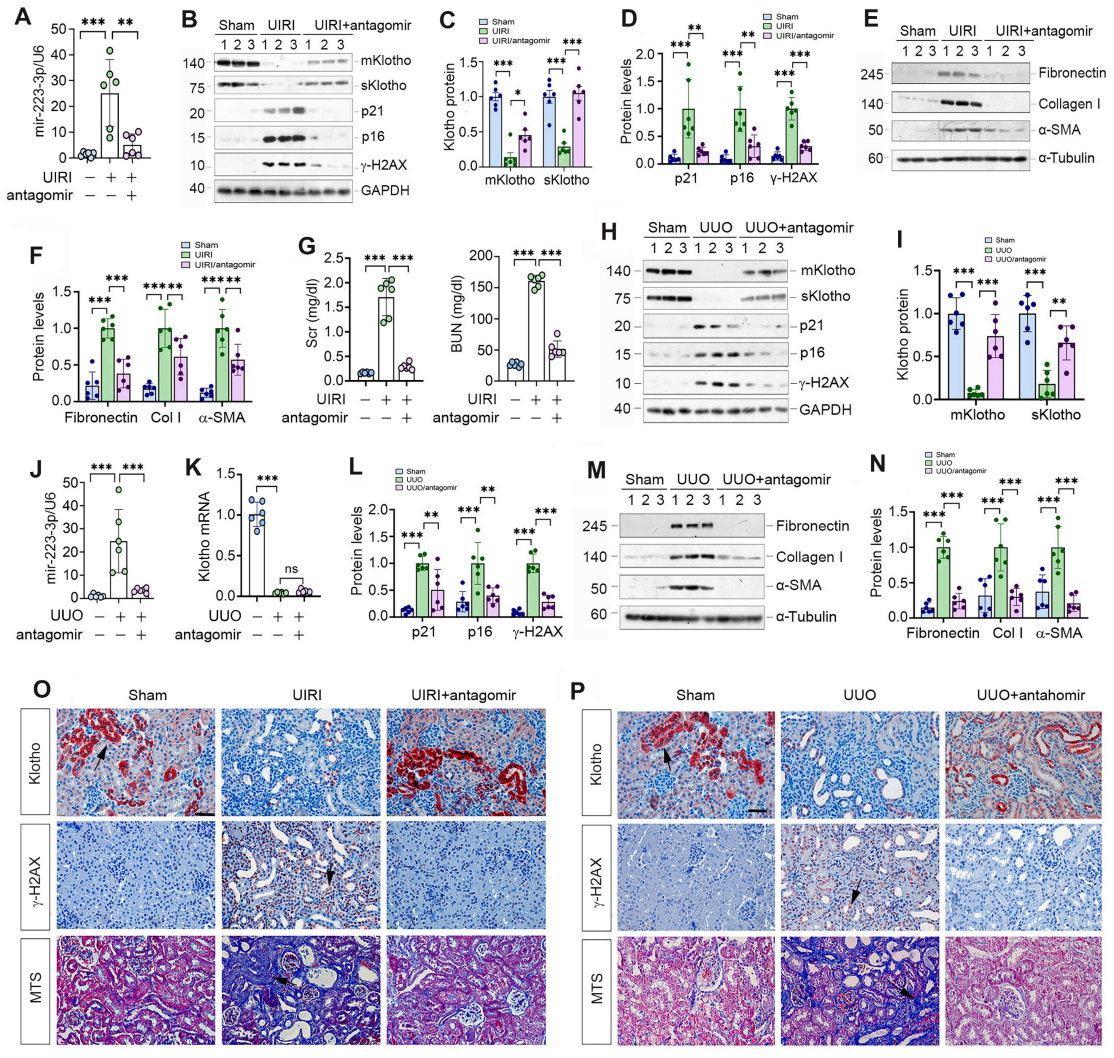
** Antagonism of miR-223-3p restores Klotho expression and ameliorates cellular senescence and kidney fibrosis *in vivo*.** (**A**) qRT-PCR analysis showed the miR-223-3p levels in the kidney of UIRI mice after injection with miR-223-3p antagomir. (**B**) Representative Western blot analyses of mKlotho, sKlotho, p21, p16 and γ-H2AX proteins in UIRI mice after injection with miR-223-3p antagomir. (**C**) Quantitative data of mKlotho and sKlotho protein levels. (**D**) Quantitative data of p21, p16 and γ-H2AX protein levels. (**E**, **F**) Representative Western blotting (**E**) and quantitative data (**F**) showed fibronectin, collagen I and α-SMA proteins in UIRI mice after injection with miR-223-3p-antagomir. (**G**) Serum creatinine (Scr) and blood urea nitrogen (BUN) of UIRI mice injected with miR-223-3p-antagomir. (**H**) Representative Western blot analyses of mKlotho, sKlotho, p21, p16 and γ-H2AX proteins in UUO mice after injection with miR-223-3p antagomir. (**I**) Quantitative data of mKlotho and sKlotho protein levels. (**J**) qRT-PCR showed renal miR-223-3p levels in different groups as indicated. (**K**) qRT-PCR showed Klotho mRNA levels in different groups as indicated. (**L**) Quantitative data of p21, p16 and γ-H2AX proteins in different groups as indicated. (**M**, **N**) Representative Western blotting (**M**) and quantitative data (**N**) showed fibronectin, collagen I and α-SMA proteins in UUO mice after injection with miR-223-3p-antagomir. (**O**) Representative immunostaining for Klotho and γ-H2AX, and MTS for collagens in UIRI mice injected with miR-223-3p-antagomir. (**P**) Representative immunostaining for Klotho and γ-H2AX, and MTS for collagens in UUO mice injected with miR-223-3p-antagomir. Scale bar, 50 µm. ^**^P < 0.01, ^**^P < 0.001 (n=6). ns, no statistical difference*.*

We explored the effect of miR-223-3p inhibition by injecting its antagomir to UIRI mice. As shown in Figure 6A-C, miR-223-3p antagomir preserved, at least partially, renal Klotho proteins. Furthermore, miR-223-3p antagomir abolished p21, p16 and γ-H2AX, fibronectin, collagen I and α-SMA in UIRI mice (Figure 6B, D-F). Antagonism of miR-223-3p also restored kidney function, as reflected by Scr and BUN levels (Figure 6G).

We also examined the effect of miR-223-3p antagomir on UUO mice. As shown in Figure 6H-K, antagomir restored renal Klotho proteins without affecting its mRNA levels. Antagonism of miR-223-3p inhibited p21, p16, γ-H2AX, fibronectin, collagen I and α-SMA in UUO mice (Figure 6H, L-N). Immunostaining for Klotho and γ-H2AX and MTS for collagens in UIRI (Figure 6O) and UUO (Figure 6P) mice produced similar results. Therefore, inhibition of miR-223-3p restored Klotho expression and mitigated cellular senescence and kidney fibrosis.

### KP1 induces Klotho expression through blocking TGF-β/Smad3/miR-223-3p axis

KP1 has been shown to block TGF-β signaling by binding to and inhibiting TβR2 26. To explore the role of TGF-β signaling in regulating miR-223-3p and Klotho expression, we used KP1, SB431542 (TβR1/2 inhibitor) and SIS3 (p-Smad3 inhibitor) to treat TGF-β1-stimulated HK-2 cells. As shown in Supplementary Figure S3A, the level of miR-223-3p was markedly upregulated by TGF-β1, which was abolished by all 3 inhibitors (Figure S3A). Notably, all 3 inhibitors repressed TGF-β1-triggered Smad3 phosphorylation in HK-2 cells (Figure S3B and C). These inhibitors also restored Klotho proteins but did not affect its mRNA levels (Figure S3B, D-E). Consistently, KP1, SB431542 and SIS3 alleviated cellular senescence caused by TGF-β1, as demonstrated by p21, p16 and γ-H2AX (Figure S3F and G). Similarly, all 3 inhibitors repressed TGF-β1-mediated fibronectin, collagen I and α-SMA expression (Figure S3H and I). These results indicate that KP1 restores Klotho expression and protects against cellular senescence by blocking the TGF-β1/Smad3/miR-223-3p signaling.

### MiR-223-3p amplifies its action through down-regulating lncRNA-TUG1

**Figure 7:**
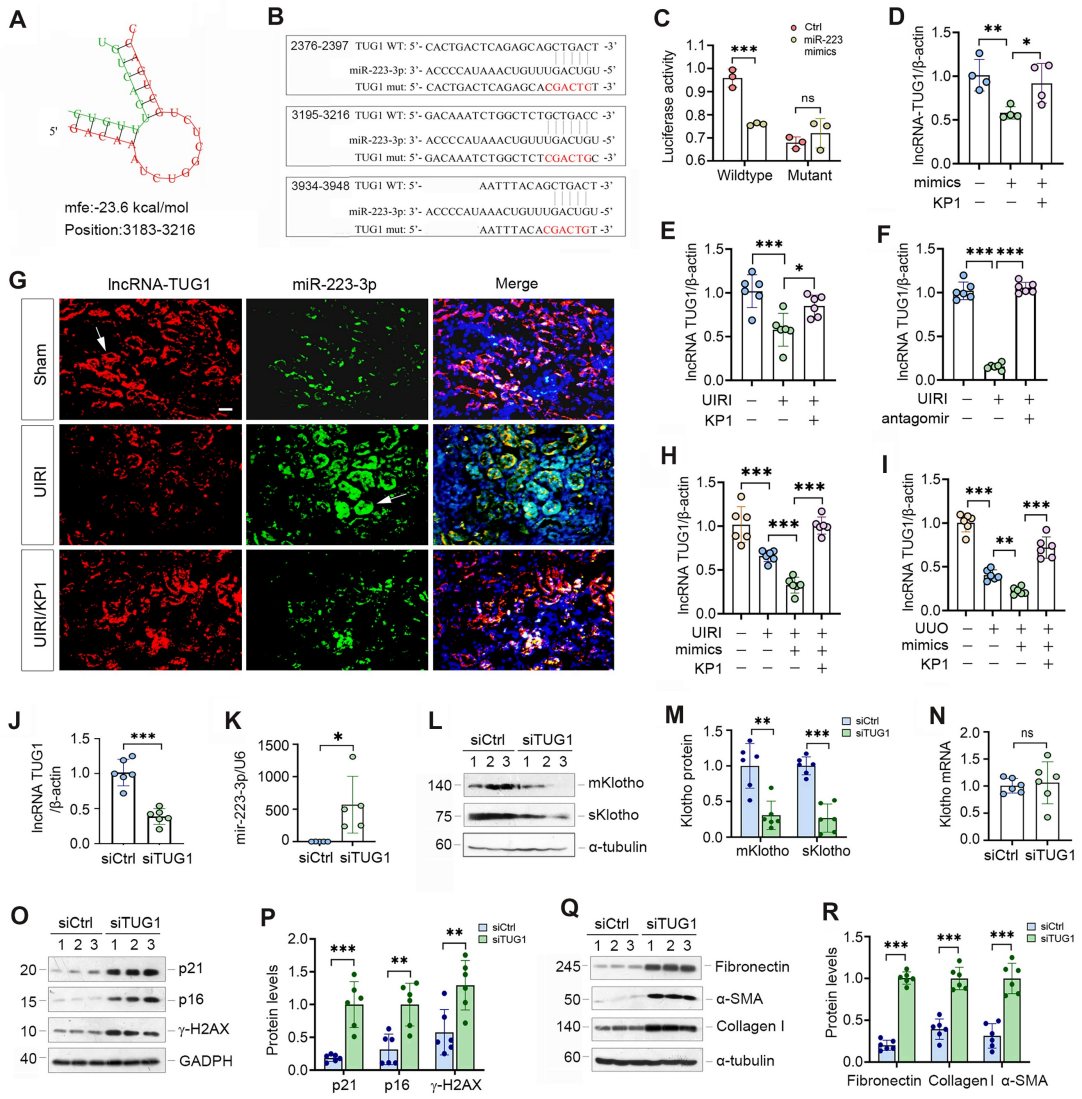
** lncRNA-TUG1 acts as a sponge for miR-223-3p and induces Klotho expression.** (**A**) Bioinformatics analysis revealed the specific binding sites of miR-223-3p and lncRNA-TUG1 predicted on RNA hybrid. (**B**) The sequences of miR223-3p and LncRNA-TUG1 binding sites. Both wild-type (WT) and mutant (mut) are present. (**C**) Relative luciferase activity in HEK-293T cells that were cotransfected with pmirGLO-h-NR152868.2-miR223-wt or pmirGLO-h-NR152868.2-miR223-mut plasmid and miR-223-3p mimics or miR-negative control (Ctrl) for 24 h. (**D**) qRT-PCR analysis of lncRNA-TUG1 levels in HK-2 cells transfected with miR-223-3p mimics in the absence or presence of KP1. (**E**, **F**) qRT-PCR analysis of lncRNA-TUG1 levels in UIRI mice injected with KP1 (**E**) or miR-223-3p antagomir (**F**). (**G**) *In situ* hybridization showed the expression and localization of lncRNA-TUG1 (red) and miR-223-3p (green) in different groups as indicated. (**H**, **I**) qRT-PCR analysis of lncRNA-TUG1 levels in UIRI (**H**) or UUO (**I**) mice injected with miR-223-3p mimics or KP1. (**J**, **K**) qRT-PCR analysis of lncRNA-TUG1 and miR-223-3p levels in HK-2 cells transfected with control siRNA (siCtrl) or TUG1-specific siRNA (siTUG1). (**L**, **M**) Representative Western blot (**L**) and quantitative data (**M**) of mKlotho and sKlotho proteins in HK-2 cells transfected with siTUG1. (**N**) qRT-PCR analysis of Klotho mRNA levels in HK-2 cells transfected with siTUG1. (**O**, **P**) Representative Western blot (**O**) and quantitative data (**P**) of p21, p16 and γ-H2AX proteins in HK-2 cells transfected with siTUG1. (**Q**, **R**) Representative Western blot (**Q**) and quantitative data (**R**) of fibronectin, collagen I and α-SMA proteins in HK-2 cells transfected with siTUG1. ^**^P < 0.01, ^***^P < 0.001. ns, no statistical difference*.*

As miRNAs and lncRNAs often interact and reciprocally regulate each other, we searched several lncRNA-miRNA interaction databases and predicted that miR-223-3p and lncRNA-TUG1 (Taurine Upregulated Gene 1) have putative binding sites (Figure 7A). We then made luciferase reporter constructs, which harbor both wild-type and mutant TUG1 sequences (Figure 7B). We carried out dual-luciferase reporter assay and found that miR-223-3p reduced the luciferase activity of wild-type but not mutant reporter (Figure 7C). Consistently, overexpression of miR-223-3p in HK-2 cells inhibited lncRNA-TUG1, which was negated by KP1 (Figure 7D). TGF-β1 inhibited lncRNA-TUG1 expression, which was negated by KP1, SB43154 or SIS3 in HK-2 cells (Supplementary Figure S4). These results suggest that lncRNA-TUG1 is subjected to regulation by TGF-β/Smad signaling.

We next assessed the expression of lncRNA-TUG1 in CKD models. LncRNA-TUG1 was decreased in UIRI kidney, but increased after treatment with KP1 or miR-223-3p antagomir (Figure 7E and F). Moreover, lncRNA-TUG1 was further reduced in UIRI and UUO mice after miR-223-3p overexpression (Figure 7H and I). However, KP1 restored lncRNA-TUG1 expression even in the presence of miR-223-3p (Figure 7H and I). These data indicate that miR-223-3p represses lncRNA-TUG1 expression, which is abolished by KP1.

To explore whether lncRNA-TUG1 can regulate Klotho and kidney injury, we knocked down lncRNA-TUG1 by using short interfering RNA (siRNA). Knockdown of lncRNA-TUG1 increased the expression of miR-223-3p (Figure 7J and K), suggesting that lncRNA-TUG1 reciprocally inhibits miR-223-3p. LncRNA-TUG1 silencing also reduced Klotho proteins (Figure 7L and M) but not its mRNA level (Figure 7N). Knockdown of lncRNA-TUG1 also increased p21, p16, γ-H2AX, fibronectin, collagen I and α-SMA proteins (Figure 7O-R). These results suggest that miR-223-3p can interact with lncRNA-TUG1 to form a competing endogenous RNA (ceRNA) network, thereby amplifying its action in regulating Klotho expression and cellular senescence.

### KP1 mitigates kidney injury triggered by lncRNA-TUG1 depletion *in vivo*

**Figure 8:**
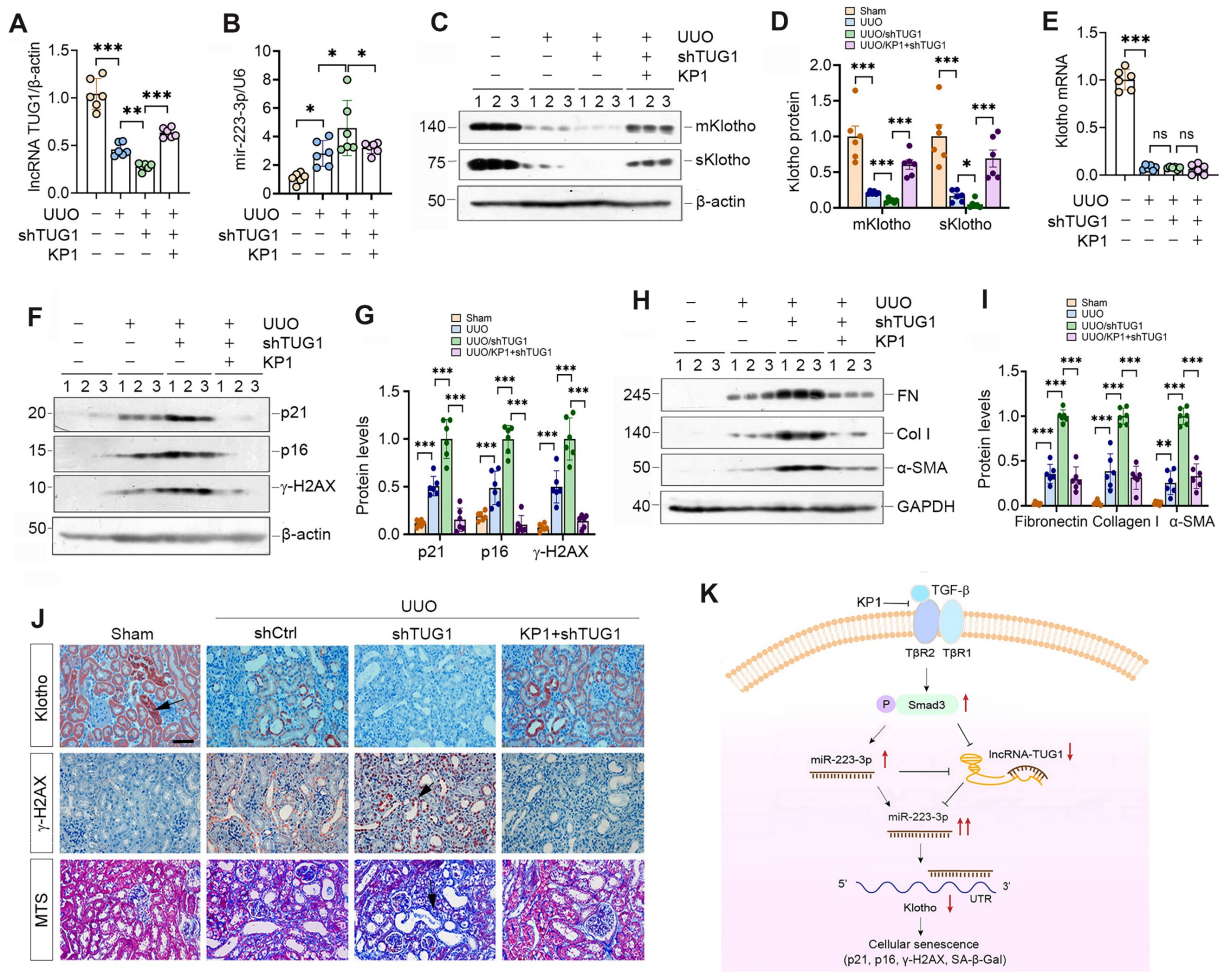
** KP1 ameliorates kidney injury by restoring lncRNA-TUG1 and Klotho expression in fibrotic kidney.** (**A**, **B**) qRT-PCR analysis of lncRNA-TUG1 (**A**) and miR-223-3p (**B**) levels in UUO mice injected with TUG1 shRNA or KP1. (**C, D**) Western blot analyses of mKlotho and sKlotho proteins in different groups as indicated. (**E**) qRT-PCR analysis of Klotho mRNA in different groups as indicated. (**F**, **G**) Western blot analyses of p21, p16 and γ-H2AX proteins in different groups of UUO mice. (**H**, **I**) Western blot analyses showed renal expression of fibronectin (FN), collagen I (Col I) and α-SMA proteins in different groups of UUO mice. (**J**) Representative immunostaining for Klotho and γ-H2AX, and MTS for collagens in different groups of UUO mice. Arrows indicate positive staining. Scale bar, 50 µm. (**K**) Schematic illustration shows that KP1 regulates cellular senescence by restoring endogenous Klotho expression via miR-223-3p and lncRNA-TUG1. KP1 restores endogenous Klotho by blocking TGF-β/Smad3/miR-223-3p pathway. lncRNA-TUG1 acts as a miR-223-3p sponge, and its loss leads to further increase in miR-223-3p and down-regulation of Klotho in CKD. ^*^P < 0.05, ^**^P < 0.01, ^**^P < 0.001.

To investigate whether lncRNA-TUG1 regulates Klotho *in vivo*, we injected plasmid containing TUG1-specific small hairpin RNA (shTUG1) into mice after UUO. As shown in Figure 8A, injection of shTUG1 resulted in a decreased lncRNA-TUG1 expression, which was abolished by KP1. However, knockdown of TUG1 increased renal miR-223-3p, whereas KP1 inhibited it (Figure 8B). Both mKlotho and sKlotho were further reduced after knockdown of lncRNA-TUG1, which was negated by KP1 (Figure 8C-D). However, neither shTUG1 nor KP1 affected Klotho mRNA (Figure 8E). KP1 also inhibited p21, p16, γ-H2AX, fibronectin, collagen I and α-SMA induced by shTUG1 (Figure 8F-J).

We extended this observation to UIRI model (Supplementary Figure S5A). Similarly, knockdown of lncRNA-TUG1 further increased miR-223-3p (Figure S5B) and decreased Klotho (Figure S5C and D), upregulated p21, p16, γ-H2AX, fibronectin, collagen I and α-SMA proteins, which were abolished by KP1 (Figure S5C, E-G). KP1 also alleviated kidney dysfunction (Figure S5H and I). Immunostaining and MTS produced similar results (Figure S5J). These results suggest that miR-223-3p and lncRNA-TUG1 work in concert, leading to Klotho loss, cellular senescence and kidney fibrosis, and all of these are alleviated by KP1 (Figure 8K).

## Discussion

Klotho, a well-known anti-aging protein with reno-protective potential, is a promising remedy for kidney disease 16. However, its usefulness in the clinic is hampered by its large size, structural complexity and high cost to produce. We recently reported the discovery of KP1, which can emulate Klotho function and alleviate CKD by inhibiting TGF-β signaling 26. In this study, we demonstrate that KP1 inhibits cellular senescence, a key event in the pathogenesis of CKD 4, 8. thereby leading to amelioration of nephropathy after UIRI and UUO. This effect of KP1 is associated with its restoration of Klotho expression at the posttranscriptional level. As depicted in Figure 8K, we identified miR-223-3p as a key player in mediating KP1 induction of Klotho expression. Furthermore, we show that miR-223-3p also binds to lncRNA-TUG1, and they mutually antagonize each other, thereby forming a feedback loop that amplifies the inhibitory action of miR-223-3p in Klotho expression. Collectively, these studies demonstrate that KP1 can inhibit cellular senescence in the fibrotic kidney by restoring Klotho expression via miRNA- and lncRNA-mediated posttranscriptional regulation. Our findings shed new light on the mechanism by which KP1 protects against nephropathy after various injuries.

Loss of Klotho is a common pathologic feature of a wide variety of CKD. As Klotho is kidney protective, its deficiency defines a permissive setting for the development and progression of nephropathies after injury. The regulation of Klotho is mainly controlled at both transcriptional and posttranscriptional levels. Many insults including high phosphate, vitamin D deficiency, angiotensin II, oxidative stress and indoxyl sulfate 30, 31 transcriptionally suppress Klotho expression by repressing Klotho promoter via complex signal networks, such as Wnt/β-catenin 15, 32, 33, TGF-β 34-36 and NF-κB 37, 38. However, the present study shows that KP1 affects neither Klotho mRNA nor its protein stability (Figure 2), suggesting that KP1 upregulation of Klotho is exclusively operated at the posttranscriptional level. Such a posttranscriptional regulation of Klotho could be carried out by non-coding RNAs, including miRNA and lncRNA 39. Of them, miRNA is particularly interesting, as it inhibits the translation of specific genes by targeting the 3'-UTR of their mRNA 40, 41. Several miRNAs such as miR-199a-5p 42, 43, miR-34a 44 and miR-126 45 have been reported to down-regulate Klotho expression. These observations prompted us to search for the key miRNA that mediates KP1 induction of Klotho protein.

Through profiling of all miRNAs in the kidney, the present study identified miR-223-3p as a key player in mediating KP1 restoration of Klotho. The expression of miR-223-3p is induced in the kidney after UIRI and UUO, but KP1 causes it to return toward baseline level. Furthermore, miR-223-3p binds to the 3'-UTR of Klotho mRNA, and represses its protein expression (Figure 3). These results are in line with numerous studies showing that miR-223-3p is up-regulated in various CKD and serves as a reliable diagnostic and prognostic marker for disease progression 46. The finding that miR-223-3p targets Klotho for suppression not only offers a novel mechanism for regulating Klotho expression but also provides a new explanation for miR-223-3p-induced kidney pathology. Although studies have shown that miR-223-3p contributes to the pathogenesis of CKD 46, the present study is the first to demonstrate that miR-223-3p promotes cellular senescence by suppressing Klotho expression. By inhibiting miR-223-3p expression, KP1 protects kidney by precisely pinning down a key point in the regulatory circuit of Klotho expression.

Another novel finding of the present study is that KP1 induces lncRNA-TUG1 expression (Figure 7). LncRNA consists of more than 200 nucleotides and is also an important player in renal pathophysiology 47, 48. Among them, lncRNA-TUG1 is known to be implicated in the pathogenesis of kidney diseases, although its function remains unclear and somewhat controversial. It has been reported that lncRNA-TUG1 exerts reno-protective effects by ameliorating fibrosis in diabetic nephropathy 49-51, attenuating ferroptosis 52 and apoptosis 53 of tubular epithelial cells in acute kidney injury, and enhancing mitochondrial function in podocytes 54. However, lncRNA-TUG1 is also implicated in facilitating the development of CKD by inducing tubular epithelial-mesenchymal transition 55, 56, exacerbating inflammation and apoptosis of tubular cells 57. In this study, we show that lncRNA-TUG1 is down-regulated in CKD, functions as miR-223-3p sponge by binding to its complementary site, and diminishes miR-223-3p-mediated Klotho suppression. Such sponge function of lncRNA-TUG1 is supported by earlier reports that several miRNAs including miR-145-5p 50, miR-144-3p 53, miR-29 58 can also bind to lncRNA-TUG1. The fact that lncRNA-TUG1 and miR-223-3p reciprocally antagonize each other underscores that they could form a double-negative feedback loop (Figure 8k), which amplifies the capacity of miR-223-3p in suppressing Klotho expression. Therefore, by repressing miR-223-3p and inducing lncRNA-TUG1, KP1 is able to restore Klotho protein at the posttranscriptional level, without affecting its mRNA abundance. This explains why KP1 can inhibit cellular senescence and ameliorates kidney injury in the fibrotic kidney, even in the condition with over-expressing miR-223-3p (Figure 5) or knockdown of lncRNA-TUG1 (Figure 8). Apart from regulating Klotho expression, whether miRNA or lncRNA-TUG1 can also directly manipulate cellular senescence remains to be investigated.

The present study has significant implications in developing future therapeutics of CKD and suggests that KP1, a peptide with 30 amino acids, is able to induce both mKlotho and sKlotho expression via epigenetic regulation mediated by miR-223-3p and lncRNA-TUG1. As such, KP1 may have a much broad actions beyond direct inhibition of TGF-β signaling as previously reported 26. By restoring Klotho expression, KP1, at least in theory, can recapitulate the full spectrum of the beneficial actions elicited by native Klotho protein. At this stage, we cannot exclude the possibility that other miRNAs besides miR-223-3p may also play a role in mediating KP1 induction of Klotho expression. Furthermore, it is also possible that KP1 may protect against nephropathies via other mechanisms independent of Klotho expression. Collectively, these possibilities suggest that KP1 may be a better remedy than other inhibitors of TGF-β signaling. Clearly, further studies are needed to address these issues.

In summary, we show herein that KP1, a newly discovered Klotho-derived peptide, inhibits cellular senescence by restoring Klotho expression. This action of KP1 is operated by miR-223-3p- and lncRNA-TUG1-mediated posttranscriptional regulation. By restoring Klotho expression, KP1 acts as a Klotho inducer and possesses a much broad spectrum of reno-protective properties, such as antioxidant, anti-senescence and stem cell protection 22. As KP1 is simple and inexpensive to make, it is hopeful that it can be translated into the clinic for CKD patients.

## Supplementary Material

Supplementary figures and tables.

## Abbreviations

CKD: chronic kidney disease
miR/miRNA: microRNAs
lncRNA: long non-coding RNAs
TUG1: taurine up-regulated 1
UIRI: unilateral ischemia-reperfusion injury
UUO: unilateral ureteral obstruction
P16: p16INK4a, cyclin-dependent kinase inhibitor 2A (CDKN2A)
P21: p21Cip1, cyclin-dependent kinase inhibitor 1 or CDK-interacting protein 1
γ-H2AX: the phosphorylated form of H2AX (H2A histone family member X)
α-SMA: α-smooth muscle actin
SA-β-gal: Senescence-associated beta-galactosidase
