# Supplementary material for: Klotho-derived peptide 1 inhibits cellular senescence in the fibrotic kidney by restoring Klotho expression via posttranscriptional regulation
Source: Theranostics. 2024 Jan 1;14(1):420–35. doi: 10.7150/thno.89105 (PMC10750200; doi:10.7150/thno.89105)
Supplement: Supplementary file 1 — Supplementary figures and tables. [file thnov14p0420s1.pdf]

Klotho-derived peptide 1 inhibits cellular senescence in the fibrotic kidney by restoring Klotho expression via posttranscriptional regulation

Xiaoyao Zhang,[1*] Li Li,[1*] Huishi Tan,[1] Xue Hong,[1] Qian Yuan,[1] Fan Fan Hou,[1,2] Lili Zhou,[1,2†] Youhua Liu[1,2†]

[1]Division of Nephrology, Nanfang Hospital, Southern Medical University, and [2]State Key Laboratory of Organ Failure Research, National Clinical Research Center of Kidney Disease, Guangdong Provincial Institute of Nephrology, Guangzhou, China

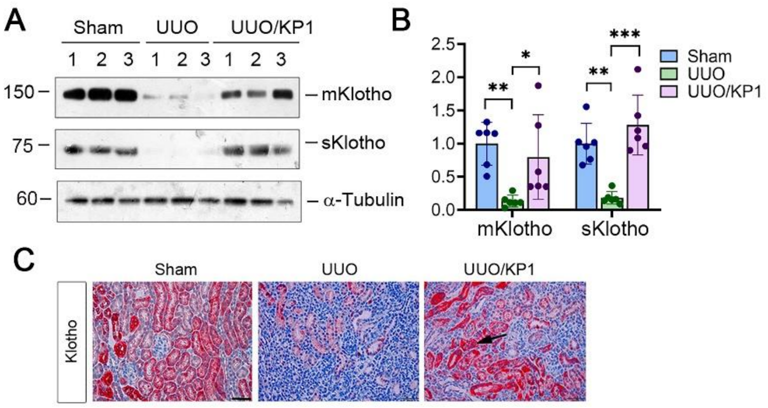

Figure S1. KP1 restores Klotho expression in the kidney of UUO mice. (**A**) Representative Western blot analyses showed renal expression of mKlotho, sKlotho proteins in UUO mice. (**B**) Quantitative data of mKlotho and sKlotho protein levels. (**C**) Representative immunostaining of Klotho in UUO mice. Arrows indicate positive staining. Scale bar, 50 µm. $^{*}P < 0.05$, $^{**}P < 0.01$, $^{***}P < 0.001$.

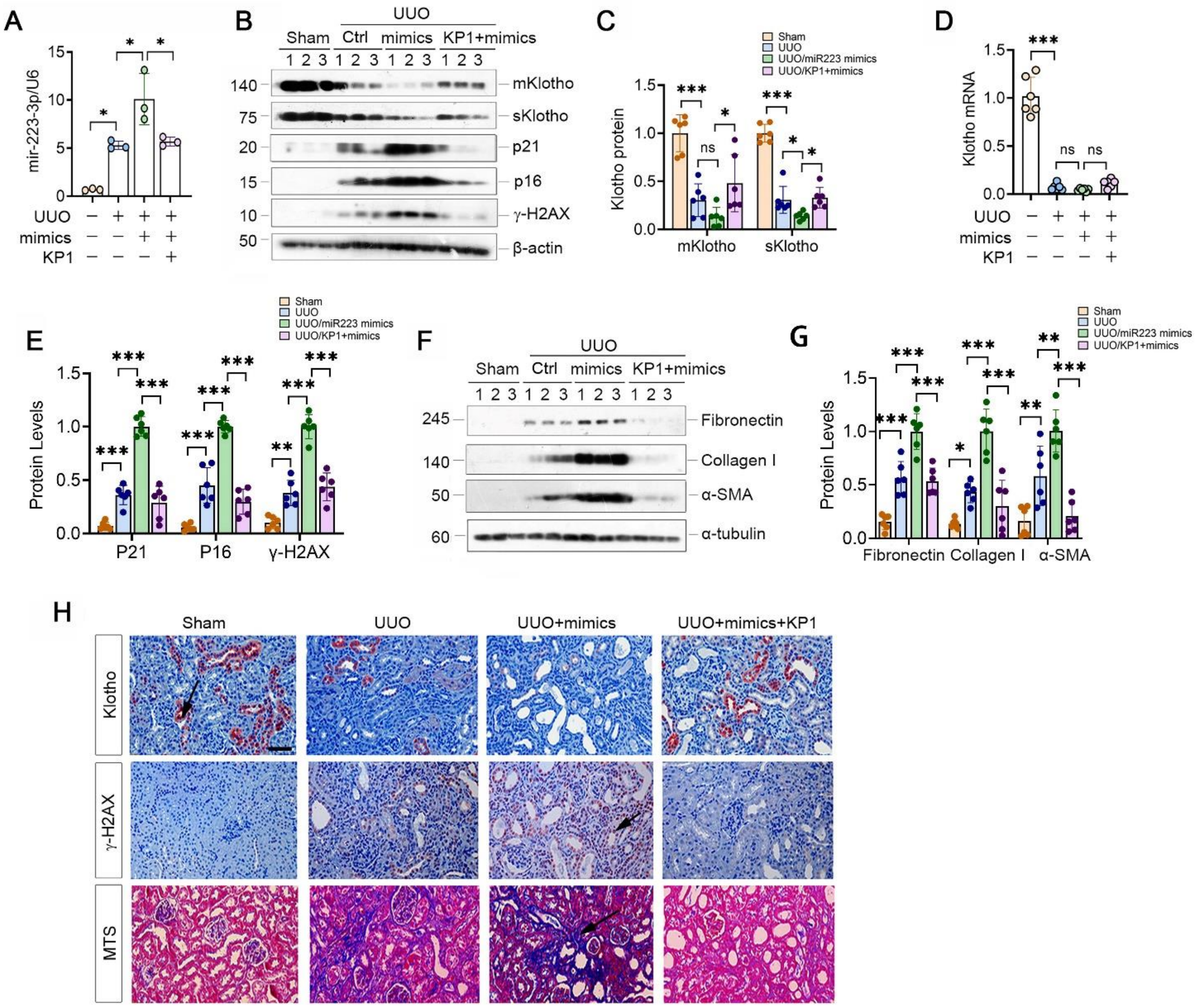

Figure S2. KP1 upregulates Klotho expression and ameliorates nephropathy by repressing miR-223-3p in UUO mice. (**A**) qRT-PCR analysis of miR-223-3p levels in different groups as indicated. (**B**) Representative Western blot analyses showed renal expression of mKlotho, sKlotho, p21, p16 and γ-H2AX proteins in UUO mice. (**C**) Quantitative data of mKlotho and sKlotho protein levels. (**D**) qRT-PCR analysis showed Klotho mRNA expression in different groups as indicated. (**E**) Quantitative data of p21, p16 and γ-H2AX proteins. (**F**, **G**) Representative Western blot (**F**) and quantitative data (**G**) showed renal expression of fibronectin, collagen I and α-SMA proteins in different groups as indicated. (**H**) Representative immunostaining of Klotho and γ-H2AX, and MTS for collagens in UUO mice. Arrows indicate positive staining. Scale bar, 50 μm. $^{*}P < 0.05$, $^{**}P < 0.01$, $^{***}P < 0.001$, n=6. ns, no statistical difference.

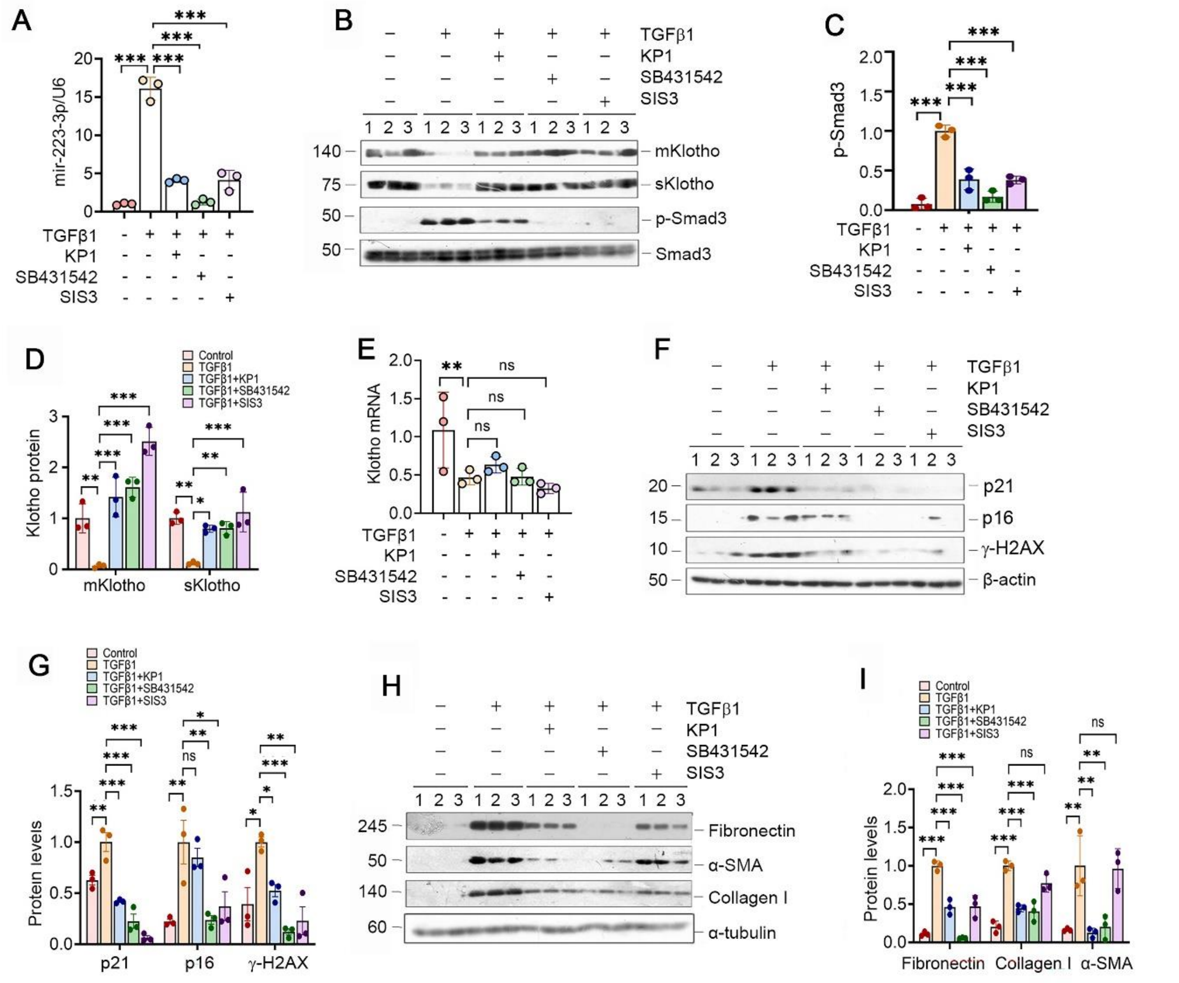

Figure S3. KP1 restores endogenous Klotho expression through inhibiting TGF-β/Smad3/miR-223-3p axis. (**A**) qRT-PCR analysis of miR-223-3p levels in TGF-β1-stimulated HK-2 cells in the absence or presence of KP1, SB431542 and SIS3, respectively. (**B-D**) Representative Western blot (**B**) and quantitative data (**C** and **D**) showed renal expression mKlotho, sKlotho and p-Smad3 proteins in TGF-β1-stimulated HK-2 cells in the absence or presence of KP1, SB431542 and SIS3. (**E**) qRT-PCR analysis of Klotho mRNA levels in different groups as indicated. (**F**, **G**) Representative Western blot (**F**) and quantitative data (**G**) showed the expression of p21, p16 and γ-H2AX proteins in TGF-β1-stimulated HK-2 cells in the absence or presence of KP1, SB431542 and SIS3. (**H**, **I**) Representative Western blot (**H**) and quantitative data (**I**) showed the expression of fibronectin, collagen I and α-SMA proteins in TGF-β1-stimulated HK-2 cells in the absence or presence of KP1, SB431542 and SIS3. $^{*}P < 0.05$, $^{**}P < 0.01$, $^{***}P < 0.001$.

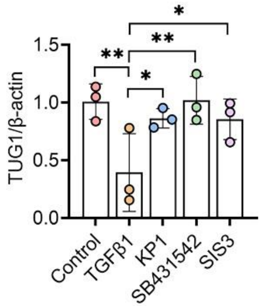

Figure S4. KP1 restores lncRNA-TUG1 expression in HK-2 cells after treatment with TGF-β1. HK-2 cells were treated with TGF-β1 in the absence or presence of KP1, SB431542 or SIS3. LncRNA-TUG1 levels were assessed by qRT-PCR. $^{*}P < 0.05$, $^{**}P < 0.01$.

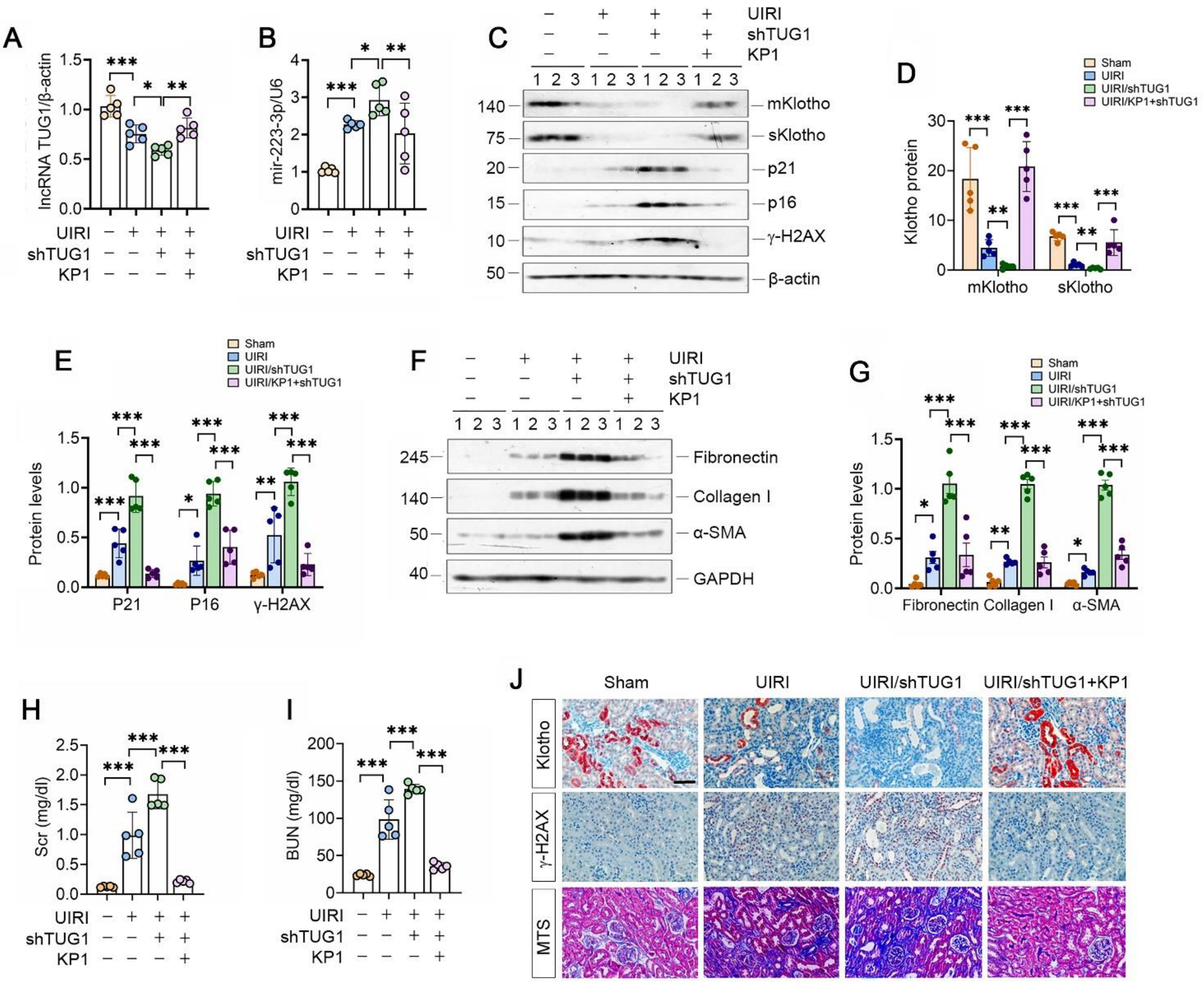

Figure S5. KP1 ameliorates kidney injury by restoring lncRNA-TUG1 and Klotho expression in the fibrotic kidney of UIRI mice. (**A**, **B**) qRT-PCR analysis of lncRNA-TUG1 (**A**) and miR-223-3p

(**B**) levels in UIRI mice injected with TUG1-shRNA in the absence or presence of KP1. (**C-E)** Representative Western blot (**C**) and quantitative data (**D**, **E**) showed renal expression of mKlotho and sKlotho, p21, p16 and γ-H2AX proteins in different groups as indicated. (**F**, **G**) Representative Western blot (**F**) and quantitative data (**G**) showed renal expression of fibronectin, collagen I and α-SMA proteins in different groups as indicated. (**H**, **I**) Serum creatinine (Scr) and blood urea nitrogen (BUN) levels in different groups as indicated. (**J**) Representative micrographs showed immunostaining for Klotho and γ-H2AX, and MTS for collagens in different groups as indicated. Scale bar, 50 μm. $^{*}P < 0.05$, $^{**}P < 0.01$, $^{***}P < 0.001$, n=5.

Table S1. The sources of antibodies used in this study

Antibodies	Company	Catalogue number	Location
**Primary antibodies**			
Anti-Fibronectin	Sigma-Aldrich	F3648	St. Louis, MO
Anti-α-SMA	Sigma-Aldrich	A2547	St. Louis, MO
Anti-α-SMA	Abcam	AB7817	Cambridge, MA
Anti-COL1A1	Boster Biological Technology	BA0325	Wuhan, China
Anti-mKlotho	R&D Systems	AF1819	Minneapolis, MN
Anti-CDKN1A/p21CIP1	ABclonal	A2691	Cambridge, MA
Anti-p16 INK4A	Santa Cruz Biotechnology	Sc-1661	Dallas, TX
Anti-Histone H2AX	ABclonal	A11463	Cambridge, MA
Anti-gamma-H2AX (phospho)	Abcam	Ab26350	Cambridge, MA
Anti-Phospho-Smad3	ABclonal	AP0727	Cambridge, MA
Anti-Smad2/3	Cell Signaling Technology	8685S	Danvers, MA
Anti-TGFβ RII	Santa Cruz Biotechnology	Sc-17791	Dallas, TX
Anti-GAPDH	Ray Antibody	RM2002	Peachtree Corners, GA
Anti-β-actin	Ray Antibody	RM2001	Peachtree Corners, GA
Anti-α-tubulin	Ray Antibody	RM2007	Peachtree Corners, GA
**Secondary antibodies**			
Peroxidase-conjugated AffiniPure Goat Anti-Mouse IgG (H+L)	Jackson Immuno-Research Laboratories.	115-035-003	West Grove, PA
Peroxidase-conjugated AffiniPure Rabbit Anti-Goat IgG (H+L)	Jackson Immuno-Research Laboratories.	305-035-003	West Grove, PA
Peroxidase-conjugated AffiniPure Goat Anti-Rabbit IgG (H+L)	Jackson Immuno-Research Laboratories.	111-035-003	West Grove, PA
Biotin-SP-conjugated AffiniPure Donkey Anti-Goat IgG (H+L)	Jackson Immuno-Research Laboratories.	705-065-147	West Grove, PA
Biotin-SP-conjugated AffiniPure Donkey Anti-Rabbit IgG (H+L)	Jackson Immuno-Research Laboratories.	711-065-152	West Grove, PA
Biotin-SP-conjugated AffiniPure Donkey Anti-Mouse IgG (H+L)	Jackson Immuno-Research Laboratories.	715-065-150	West Grove, PA

Table S2. The nucleotide sequences of the primers used for qPCR

Gene	Primer Sequences 5' to 3'	
	**Forward**	**Reverse**
mmu-Klotho	AAAGTAGACGGGGTTGTAGCC	CGGTAGAAGTGCAGAACCGT
hsa-Klotho	GTGCGTCCATCTGGGATACG	TGTCGCGGAAGACGTTGTT
has-miR-223-3p	UGUCAGUUUGUCAAAUACCCCA	
mmu-miR-223-3p	UGUCAGUUUGUCAAAUACCCCA	
hsa-TUG1	TAGCAGTTCCCCAATCCTTG	CACAAATTCCCATCATTCCC
mmu-TUG1	GAGACACGACTCACCAAGCA	GAAGGTCATTGGCAGGTCCA

Table S3. The nucleotide sequences of Oligo DNA/RNA used in this study

RNA	Sequences 5'to 3'
hsa-miRNA inhibitor N.C.	CAGUACUUUUGUGUAGUACAA
hsa-miR-223-3p inhibitor	UGGGGUAUUUGACAAACUGACA
hsa-miRNA mimics N.C.	sense: UUCUCCGAACGUGUCACGUTT
	antisense: ACGUGACACGUUCGGAGAATT
hsa-miR-223-3p mimics	sense: UGUCAGUUUGUCAAAUACCCCA
	antisense: GGGUAUUUGACAAACUGACAUU
siRNA N.C.	sense: UUCUCCGAACGUGUCACGUTT
	antisense: ACGUGACACGUUCGGAGAATT
siTUG1-homo-1144	sense: GCUACAACUAUCUUCCUUUTT
	antisense: AAAGGAAGAUAGUUGUAGCTT
siTUG1-homo-222	sense: GAGCAGGCUAUCAGAAUAATT
	antisense: UUAUUCUGAUAGCCUGCUCTT
siTUG1-homo-3739	sense: GCCUCUAUUCCUGUAUGUATT
	antisense: UACAUACAGGAAUAGAGGCTT
siRNA-TUG1-4134	sense: GGACAAACUUAUCUCUCAUTT
	antisense: AUGAGAGAUAAGUUUGUCCTT
mmu-miRNA-mimics-NC	sense: AATTCGTTCTCCGAACGTGTCACGTGTTTTGG
	CCACTGACTGACACGTGACATTCGGAGAAA
	antisense: CCGGTTTCTCCGAATGTCACGTGTCAGTCAGT
	GGCCAAAACACGTGACACGTTCGGAGAACG
mmu-miR-223-3p mimics	sense: TGCTGTGTCAGTTTGTCAAATACCCCAGTTTTGG
	CCACTGACTGACTGGGGTATGACAAACTGACA
	antisense: CCTGTGTCAGTTTGTCATACCCCAGTCAGTCAGT
	GGCCAAAACTGGGGTATTTGACAAACTGACAC
mmu-miR-223-3p antagomir	UGGGGUAUUUGACAAACUGACA (3'cholesterol modification)
mmu-shRNA-NC	sense: CACCGTTCTCCGAACGTGTCACGTTTCAA
	GAGAACGTGACACGTTCGGAGAACTTTTTTG
	antisense: GATCCAAAAAAGTTCTCCGAACGTGT
	CACGTTCTCTTGAAACGTGACACGTTCGGAGAAC
mmu-shRNA-TUG1-2335	sense: CACCGCATATTGTCAACCTGTTTGCTTCAAGA
	GAGCAAACAGGTTGACAATATGCTTTTTTG
	antisense: GATCCAAAAAAGCATATTGTCAACCTGTTTGCT
	CTCTTGAAGCAAACAGGTTGACAATATGC
mmu-shRNA-TUG1-500	sense: CACCGCACTGTCACTGGGAACTTGATTCAAG
	AGATCAAGTTCCCAGTGACAGTGCTTTTTTG
	antisense: GATCCAAAAAAGCACTGTCACTGGGAACTTGA
	TCTCTTGAATCAAGTTCCCAGTGACAGTGC
mmu-shRNA-TUG1-3525	sense: CACCGCTACTGCAATTAGACTAACTTTCAAG
	AGAAGTTAGTCTAATTGCAGTAGCTTTTTTG
	antisense: GATCCAAAAAAGCTACTGCAATTAGACTAAC

		TTCTCTTGAAAGTTAGTCTAATTGCAGTAGC
mmu-shRNA-TUG1-277	sense:	CACCGGAGGTTCATAAAGTACATGCTTCAAGAGAGCATGTACTTTATGAACCTCCTTTTTTG
	antisense:	GATCCAAAAAGGAGGTTCATAAAGTACATGCTCTCTTGAAGCATGTACTTTATGAACCTCC

Table S4. The plasmid vectors of Oligo DNA/RNA and control vectors used in this study

Oligo DNA/RNA	Plasmid vectors	Control vectors
hsa-miR-223-3p inhibitor	microRNA inhibitors	hsa-miRNA inhibitor N.C.
hsa-miR-223-3p mimics	Double-stranded microRNA mimics	hsa-miRNA mimics N.C.
mmu-miR-223-3p antagomir	miR-DownTM antagomir	mmu-miRNA antagomir N.C.
mmu-miR-223-3p mimics	CMV/EGFP/miR/Blasticidin	mmu-miRNA-mimics-NC
siTUG1-homo-1144	Double-stranded siRNA	siRNA N.C.
siTUG1-homo-222	Double-stranded siRNA	siRNA N.C.
siTUG1-homo-3739	Double-stranded siRNA	siRNA N.C.
siTUG1-homo-4134	Double-stranded siRNA	siRNA N.C.
mmu-shRNA-TUG1-2335	pGPU6/GFP/Neo	pGPU6/GFP/Neo shNC
mmu-shRNA-TUG1-500	pGPU6/GFP/Neo	pGPU6/GFP/Neo shNC
mmu-shRNA-TUG1-3525	pGPU6/GFP/Neo	pGPU6/GFP/Neo shNC
mmu-shRNA-TUG1-277	pGPU6/GFP/Neo	pGPU6/GFP/Neo shNC
pmirGLO-h-KL-miR223-wt	pmirGLO Vector	pmirGLO-h-miR223-mut
pmirGLO-h-NR152868.2-miR223-wt	pmirGLO Vector	pmirGLO-h-NR152868.2-miR223-mut
